# Plastic and Reconstructive Surgery in the Treatment of Oncological Perineal and Genital Defects

**DOI:** 10.3389/fonc.2015.00212

**Published:** 2015-10-08

**Authors:** Rebekka Brodbeck, Raymund E. Horch, Andreas Arkudas, Justus P. Beier

**Affiliations:** ^1^Department of Plastic and Hand Surgery, University Hospital of Erlangen, Friedrich-Alexander-University Erlangen-Nuernberg, Erlangen, Germany

**Keywords:** interdisciplinary surgery, exenteration, VRAM flap, perineal reconstruction, microsurgical free flap

## Abstract

Defects of the perineum may result from ablative procedures of different malignancies. The evolution of more radical excisional surgery techniques resulted in an increase in large defects of the perineum. The perineogenital region *per se* has many different functions for urination, bowel evacuation, sexuality, and reproduction. Up-to-date individual and interdisciplinary surgical treatment concepts are necessary to provide optimum oncological as well as quality of life outcome. Not only the reconstructive method but also the timing of the reconstruction is crucial. In cases of postresectional exposition of e.g., pelvic or femoral vessels or intrapelvic and intra-abdominal organs, simultaneous flap procedure is mandatory. In particular, the reconstructive armamentarium of the plastic surgeon should include not only pedicled flaps but also free microsurgical flaps so that no compromise in terms of the extent of the oncological resection has to be accepted. For intra-abdominally and/or pelvic tumors of the rectum, the anus, or the female reproductive system, which were resected through an abdominally and a sacrally surgical access, simultaneous vertical rectus abdominis myocutaneous (VRAM) flap reconstruction is recommendable. In terms of soft tissue sarcoma of the pelvic/caudal abdomen/proximal thigh region, two-stage reconstructions are possible. This review focuses on the treatment of perineum, genitals, and pelvic floor defects after resection of malignant tumors, giving a distinct overview of the different types of defects faced in this region and describing a number of reconstructive techniques, especially VRAM flap and pedicled flaps like antero-lateral thigh flap or free flaps. Finally, this review outlines some considerations concerning timing of the different operative steps.

## Introduction

Defects of the perineum usually result from ablative procedures of different malignancies, such as gynecological (cervix, vagina, endometrial), urological (urinary bladder, prostate), and colorectal (anal and rectal carcinoma) tumors. The evolution of more radical excisional surgery techniques resulted in an increase in large defects of the perineum (1). The perineogenital region *per se* has many different functions for urination, bowel evacuation, sexuality, and reproduction (2), so extensive resection in this region results often in functional deficits.

Pelvic surgery is characterized by a complex anatomy, involvement of different organs and microbial environment of this region. Plastic-reconstructive measures like simultaneously used skin grafts, pedicled, or free flaps avoid different complications or reduce their incidence, such as chronic wound healing disorders and chronic secretion of intrapelvic or peritoneal wound cavities (3, 4). For locally advanced primary or recurrent rectal cancer invading the urine bladder or prostate, pelvic exenteration is often the only treatment, with is potentially curative (5–7). Radical surgery completely resects all malignant disease, often including the complete or at least large parts of pelvic viscera, vessels, muscles, ligaments, or pelvic bone. In modern concepts of advanced oncological surgery, survival is not the only consideration; quality of life has to be taken into account (8). Up-to-date individual and interdisciplinary surgical treatment concepts are necessary to provide optimum oncological as well as quality of life outcome (3, 9).

This review focuses on the treatment of perineum, genitals, and pelvic floor defects after resection of malignant tumors, giving a distinct overview of the different types of defects faced in this region and describing a number of reconstructive techniques. Finally, this review outlines some considerations concerning timing of the different operative steps.

Literature about pelvic reconstruction in particular is very rare and review articles or larger case series are absent, hence only case reports have been published. There is only scarce literature with sufficient evidence on this topic, apart from some single case reports and a few case series, this will be critically discussed in the following review.

## Pelvic Region: Extra- and Intrapelvic Tumors

In the pelvic and the inguinal/proximal femoral region, there are essentially two various tumor entities to discuss. Their anatomical localization and extension require very different treatment concepts. In course of this, both initial multidisciplinary team approach and form of reconstructive measures have been adapted to it (3, 8). On the one hand, there are mainly intra-abdominal and pelvic tumors, which are mostly low rectal carcinomas and deep infiltrating anal carcinomas (10), as well as far advanced gynecological tumors [e.g., vulval cancer (11) or cervix cancer (3)]. On the other hand, there are soft tissue sarcomas of the pelvis, the caudal abdomen, and the proximal femoral region (12).

Despite this, there are many other indications, like congenital defects, infections, trauma, lymphedema, and other uncommon problems, e.g., transsexuality (2) requiring reconstructive surgery in the pelvic region, which are not subject of this review.

Different reconstruction methods are available for the above-mentioned malignancies. An overview of the most common pedicled (Table 1) and free flaps (Table 2) for reconstruction of the perigenital region is shown in Tables 1 and 2.

**Table 1:** **The most useful pedicled flaps for defect reconstruction of the pelvic region [modification of Beier et al. (3) and Das Gupta et al. (13)]**.

Pedicled flaps	Vascular supply	Region of defect reconstruction
Gluteus muscle flap	Superior gluteal artery/inferior gluteal artery	Sacral
SGAP/IGAP flap	Superior/inferior gluteal artery perforator	Sacral
TRAM flap	Inferior epigastric artery	Pelvic floor
VRAM flap	Inferior epigastric artery	Pelvic floor
Groin flap	Medial circumflex femoral artery/superficial circumflex iliac artery	Perineal
SCIP flap	Superficial circumflex iliac artery perforator	Inguinal
Gracilis muscle flap	Medial circumflex femoral artery	Perineal
Pudendal flap	External pudendal artery	Perineal
Tensor fascie latae flap	Lateral circumflex femoral artery	Perineal
Rectus femoris muscle flap	Lateral circumflex femoral artery	Ischial
Vastus lateralis muscle flap	Lateral circumflex femoral artery	Ischial/Perineal

*SGAP, superior gluteal artery perforator; IGAP, inferior gluteal artery perforator; TRAM, transversal rectus abdominis myocutaneous flap; VRAM, vertical rectus abdominis myocutaneous flap; SCIP, superficial circumflex iliac artery perforator*.

**Table 2:** **The most useful microsurgical free flaps for defect reconstruction of the pelvic region [modification of Beier et al. (3) and Das Gupta et al. (13)]**.

Vascular system	Microsurgical free flaps
Subscapularis artery	Latissimus dorsi muscle flap (thoracodorsal artery) Scapular/Parascapular flap (circumflex scapula artery) Serratus anterior muscle flap (serratus branch of thoracodorsal artery) Combinations
Inferior epigastric artery	Vertical rectus abdominis myocutaneous (VRAM) flap expanded VRAM Transversal rectus abdominis myocutaneous (TRAM) flap or deep inferior epigastric perforator (DIEP) flap
Lateral circumflex femoral artery	Antero-lateral thigh (ALT) flap (descending branch) Tensor fascie latae (TFL) flap (ascending branch) Combinations with rectus femoral muscle, lateral vastus muscle, etc.

In most of the cases, primary closure of perineal defects is not possible. Skin grafts are suboptimal in the perigenital area due high bacterial load in this region, frequently leading to graft loss, prolonged healing resulting in unsatisfactory scar quality and contractures that may affect urination or coitus (14). Over the past decades, flap reconstruction has replaced these techniques in the vast majority of cases. An ideal flap should provide soft-tissue volume to close dead space in pelvis and the skin island should replace resected perineal skin (15). These flaps will be described and discussed in detail for the two different groups of malignancies in the pelvic regions in the following paragraph.

## Plastic-Reconstructive Measures of Rectal and Anal Carcinomas

Modern treatment of rectal and anal carcinomas includes a multimodal therapy concept. Preoperative neoadjuvant radio-chemotherapy has become standard treatment for rectal cancer and has been shown to downstage tumors (15, 16), before radical aggressive surgery is applied to achieve a lasting cure (8, 17). Despite the concept that multidisciplinary team approach is identically for both entities, the surgical therapy concept itself is very different, associated with the plastic-reconstructive possibilities (3).

**Figure 1:**
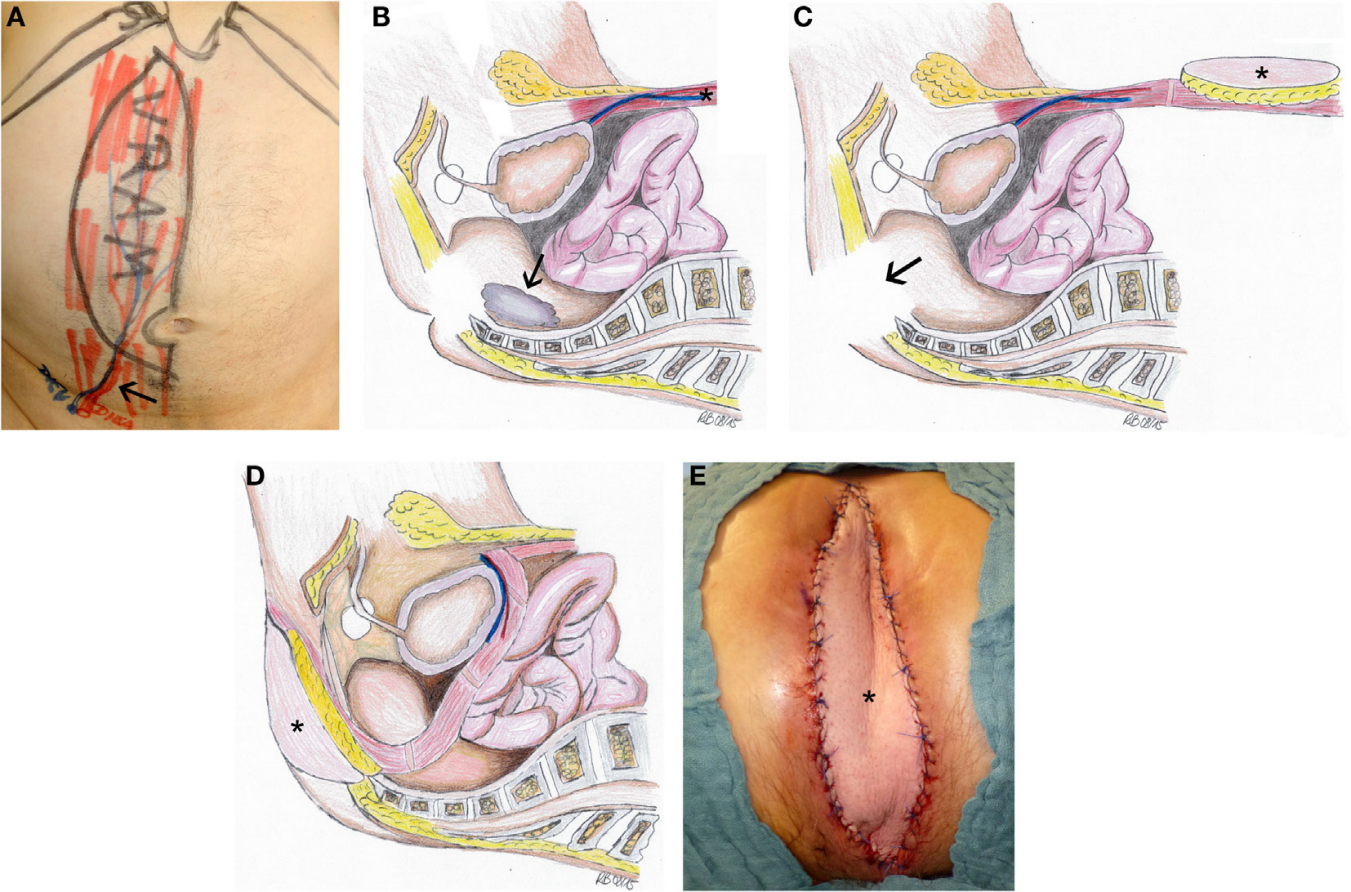
**Defect reconstruction after resection of a rectal carcinoma using VRAM flap illustrated by intraoperative photographs and schematic drawings of the surgical technique**. **(A)** Preoperative marking for VRAM-flap procedure with the planned skin paddle and location of the ostomy performed on the day before surgery. Black arrow marks the flap pedicle. **(B)** The operation involves a two-part procedure with an anterior abdominal dissection first, which is followed by a second step with perineal tumor excision (black arrow) in prone position. We first ensure the viability of the deep inferior epigastric vessels before we proceed with the flap raising. The design of the flap and the size of the skin paddle are then planned according to the prospective perineal and pelvic defect. The skin island is placed vertically over the rectus muscle. The rectus muscle is dissected cranially from the costal arch. In the prone phase, tumor excision (black arrow) had been completed **(C)**. The flap (black asterisk) is then flipped and rotated at 180° into the pelvic cavity so that the skin paddle closes the defect **(D)**. Intraoperative view with VRAM flap (black asterisk) inserted to reconstruct perineal defect **(E)**.

For intra-abdominal and/or pelvic tumors of the rectum, the anus or the female reproductive system, which were resected through an abdominal and a perineal surgical access (18), simultaneous flap reconstruction is recommendable. The goal is not only a perineal and/or sacral defect reconstruction but also an intrapelvic sealing, as well as a vaginal partial reconstruction if necessary; both is facilitated by the vertical rectus abdominis myocutaneous (VRAM) flap (Figure 1) (8, 13, 19). To achieve an oncological safe situation, aggressive surgery must be implemented and performing pelvic exenteration with “en bloc” resection of multiple pelvic structures is necessary (8, 9, 18). After abdominoperineal extirpation, often a large intrapelvic cavity remains, perineal wound complications including wound dehiscence and longtime of secretion occur, even according to radiotherapy. Studies have shown that the VRAM flap is a reliable and safe method for pelvic reconstruction in patients with advanced disease requiring pelvic exenteration and radiation, with relatively low rate of donor and recipient site complications (20–22).

During the last years, important advances in generation of vascularized tissue engineering have been achieved (23). However, until today flap surgery still remains the gold standard for plastic-reconstructive treatment of oncological defects (24). The immediately/simultaneously used transpelvic VRAM flap has several advantages: first of all, the VRAM flap is a very safe and robust flap and relatively easy to technical perform, when necessary plastic-reconstructive expertise exists. Furthermore, the vascular supply of the deep inferior epigastric vessel is constant. Using the VRAM flap as a transpelvic flap not only allows reconstruction of perineal and perigenital skin defects, but also enables obliteration of the sacral cavity (22, 25, 26). Principally, alloplastic and biological matrices have also been used to avoid a herniation of the small bowel, but these techniques are correlated with a significant risk for foreign body reaction, and are prone to infections and formation of chronic fistula, especially if non-absorbable matrices have been used in a radiated field (27, 28). Furthermore, a vascularized muscle flap can reliably fill dead space in the pelvis and can even help to cure local infection (29).

Vertical rectus abdominis myocutaneous flap can also be used for reconstruction of the vagina, when part of the vagina are infiltrated by the tumor and need to be excised. Therefore, the unilateral caudally pedicled VRAM flap can reconstruct half of circumference of the vagina (8). Moreover, vaginal fistula development can be avoided, because of the sealing effect and the well-vascularized tissue over the sutured vaginal stump (30).

A situation after multiple abdominal surgeries represents a major challenge for VRAM flap implementation. On the one hand, the deep inferior epigastric vascular supply may not be available anymore; on the other hand, significant scarring complicates the preparation. Careful preparation and experience of the surgeon permit the implementation of a VRAM flap even in these cases; furthermore, assessment of the deep inferior epigastric vessels through computer angiography is recommended.

Vertical rectus abdominis myocutaneous flaps can also be desepithelialized, which allows to obliterate larger dead space volumes and adjustment of the skin paddle to smaller skin defects, no bulky perineal skin surface and a shorter suture line is achieved. Vascularized dermis at the wound base seems to be associated with a rapidly healing, even in an irradiated field (31, 32).

The most common defects of tumors in this region are caused by rectal and anal carcinomas. In addition, excision of other tumors necessitates a resection of the neighboring skin-/soft tissue, which requires a reconstruction during the same operation. This includes, e.g., extensive gynecological and bladder carcinomas. In case of resection of pelvic bone, VRAM flap can be transferred anteriorly to the symphysis instead of the transpelvic course (33). Two-stage reconstruction has some disadvantages in such cases. Secondary reopening of the abdomen carries a lot of risks, like discrete intestinal loops that are easily being injured during dissection, e.g., interim negative wound pressure therapy is a possible option, but carries the risk for chronic fistulas through the continuous negative pressure (34), hence single-stage reconstruction is strongly recommended under these circumstances.

Summarizing, we suggest that VRAM flap is a particularly suitable method for pelvic reconstruction in patients with advanced colorectal cancer disease requiring pelvic exenteration (20–22).

## Plastic-Surgical Measures of Sarcomas of the Pelvic Region

In terms of soft tissue sarcoma of the pelvic/caudal abdomen/proximal thigh region, two-stage reconstruction is possible. Until the final histopathological results, negative wound pressure therapy can be used after tumor resection. Additional resections can be performed until histopathological R0-margins are achieved. Nevertheless simultaneous tumor resection and defect reconstruction can be useful (35).

In general, perineal soft tissue tumors are rare, so optimizing their management and outcome of treatment are still subject to investigation (12). General guidelines have been published, although the histological types are very variable and the locations mostly very complex and various (36, 37). Soft tissue sarcoma requires individual treatments, because optimal local control prevents deaths, related to local progression (38). The soft tissue sarcoma tumor size is often very large, because these tumors grow often without symptoms in the ischioanal fossa (12). In case of chemo- or radiosensitive subtypes, neoadjuvant radio-chemotherapy should be discussed (36).

Treatment of arrosion hemorrhage and exposed osteosynthesis implants are urgent indications of simultaneous reconstructions (39). Another indication for simultaneous defect reconstruction is exposition of vulnerable structure like nerves or vessels, e.g., negative wound pressure therapy (possibly using an additional silicon membrane beneath the sponge for protection of underlying structures) may be applied in between, since harm of nerves by negative pressure therapy has not been reported so far. Nevertheless, the risk especially for infection of expanded defects even after radio-chemotherapy is very high; therefore, simultaneous defect reconstruction is recommended.

However, timing of reconstruction is very challenging. To achieve a long-lasting cure, thorough examination of the patient and his local findings, as well as the radiologic findings, has to be evaluated in a multidisciplinary approach. No compromise in terms of the extent of the oncological resection has to be accepted, and the extent of the defect decides the treatment regime as a simultaneous vs. a two-stage defect reconstruction (3).

**Figure 2:**
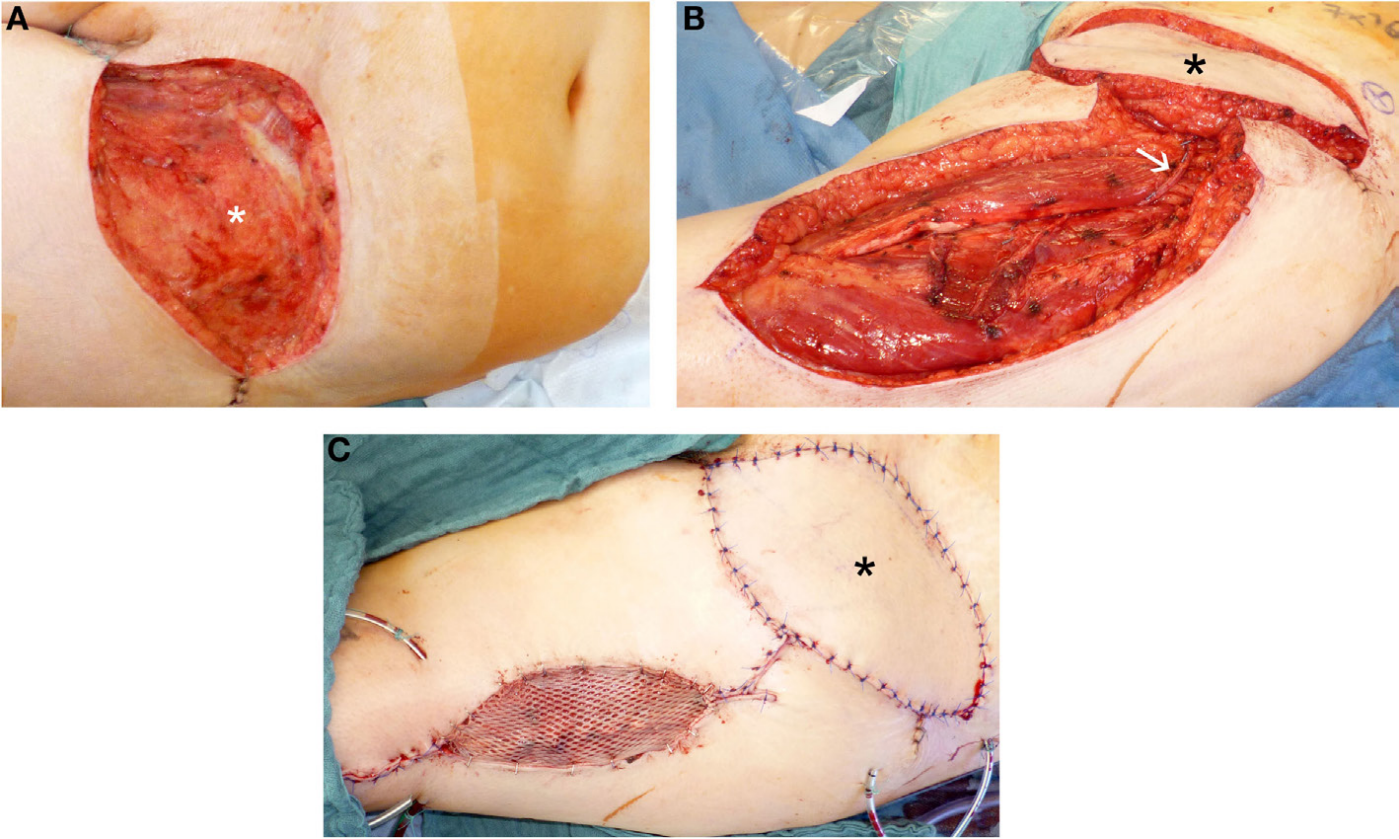
**Defect reconstruction at groin after resection of a dermatofibrosarcoma protuberans using caudal pedicled ALT flap**. **(A)** Extent of groin defect after resection (black asterisk) of a dermatofibrosarcoma protuberans. **(B)** Intraoperative view after dissection of ALT flap and rotation into the groin defect. For typical manner of harvesting ALT flap, ALT perforator is localized between the central to lower third of the ALT flap area after skin incision and initial preparation. White arrow shows the ALT perforator. **(C)** Intraoperative view at the end of the operation with ALT flap (black asterisk) and skin graft at donor site to reconstruct groin defect.

As an example for two-stage defect reconstruction and using a pedicled antero-lateral thigh (ALT) flap, Figure 2 shows the case of a patient with dermatofibrosarcoma protuberans at the left groin. Negative wound pressure therapy was applied until the histopathological R0-result was confirmed. For defect reconstruction (Figure 2A), a caudally pedicled ALT flap was used (Figures 2B,C).

All in all, in cases of reconstructions after sarcoma resection in the pelvic region, we recommend a two-stage reconstruction where possible, i.e., when no vulnerable structures are exposed after tumor resection. Many factors like size and character of the defect have to receive attention, but often a pedicled flap like ALT flap or VRAM flap could be used (3, 40).

## Combined Intra- and Extrapelvic Defects

Combined intrapelvic organ defects (e.g., chronic bladder fistulas) and concurrent abdominal skin defects (e.g., with abdominal skin fistulas and/or unstable scars or skin grafts) are very challenging. In particular, due to side effects of radiotherapy, skin grafts are prone to complications. Most times the defect may initially underestimated and skin grafts are applied, which need to be replaced during the further course by vascularized tissue/flaps.

As an example for such postoperative complication, a vesico-cutaneous fistula of the caudal rectus abdominis muscle after sarcoma resection and radiotherapy may result, which is very difficult to treat (41). For treatment of this rare entity, microsurgical free flap transplantation can become necessary, especially to avoid an additional weakening of the abdominal wall using the contralateral rectus abdominal muscle. A combined bipedicular latissimus dorsi/anterior serrate flap is capable of covering the intrapelvic defect (bladder vault using the part of serratus flap) as well as the abdominal wall defect cranial to the symphysis (using the part of latissimus dorsi flap) (42).

Reconstruction of combined intra- and extrapelvic defects is always very challenging. The literature describes no patent remedy, so we suggest for treatment of this rare entity microsurgical free flap transplantation (42, 43).

## Secondary Treatment of Perigenital Defects

Sometimes patients present themselves, secondary or after complications have occured, like recurrent abscending or phlegmonous infections of the pelvis, persistent severe secretion out of chronic sacral cavities or fistulas in prostate/vaginal/urine bladder region. Treatment of such sequelae is technically very challenging and connected to a severe risk profile. A reopening of the abdominal access for VRAM flap is difficult, because of a high risk for injury of adherent small intestine or fibrosis as well as stenosis of the pelvic entry. Another problem is loss of both inferior epigastric vessels. In these cases, other pedicled regional flaps should be used for defect reconstruction (15).

To cover posterior defects, gluteal flaps are useful (1); for ventral defects, pudendal flaps (44) and groin flaps (45) should be mentioned. Similarly, the use of lateral vastus muscle flap (46) and ALT flap (15) for defect reconstruction after pelvic exenteration has been described. Myocutaneous gluteal flaps are mainly used as rotation – or as VY advancement – flaps (47), while the gracilis muscle flap is mainly used as proximally pedicled muscle flap (48). Complications result most from chronic lymphfistulas or lymphedema at femoral or groin region. Gluteal perforator flaps (SGAP and IGAP) do not affect the motor function and minimize the donor-side morbidity (49), thus, offering a technically more advanced solution with relatively low donor site morbidity.

A successful defect reconstruction including in particular a sufficient filling of presacral cavity requires an acceptable intrapelvic access using pedicled gluteal muscle flaps. Sometimes sacral or coccygeal bone has been resected (50).

**Figure 3:**
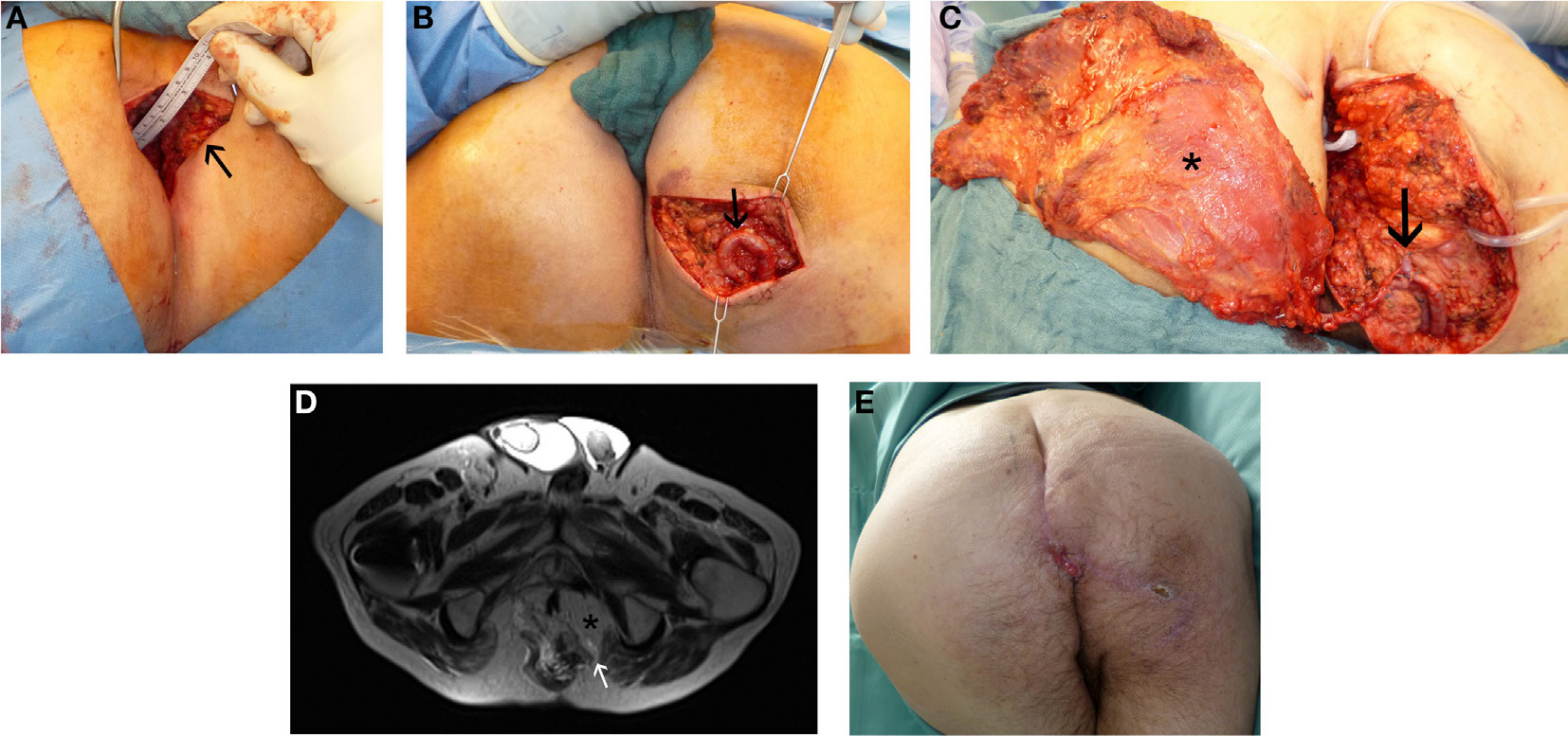
**Perineal defect reconstruction after resection of a chronic sacral cavity with fistulas using a microsurgical free “buried” latissimus dorsi flap and an arterio-venous loop**. In history, after radio-chemotherapy a rectal carcinoma had been resected. **(A)** Preoperative presentation of chronic sacral fistula (black arrow). **(B)** Situs after dissection of the arterio-venous loop (black arrow). **(C)** Intraoperative view at anastomosed latissimus dorsi flap (black asterisk) at the loop (black arrow). **(D)** MR-Angiography with microsurgical free “buried” latissimus dorsi flap (black asterisk) inserted to reconstruct perineal defect and imaging of the consistent arterio-venous loop (white arrow). **(E)** Result 1 month postoperatively.

Secondary sealing itself is very challenging, if chronic sacral cavities or recurrent pelvic infections are being observed. An access to and sufficient filling of presacral dead space is difficult to achieve in a secondary perineal approach. In such cases, sufficient three-dimensional defect reconstruction often is not possible without microsurgical free flaps. These could be anastomosed to local blood vessels, like gluteal vessels (51), femoral vessels, or iliac vessels after an arterio-venous loop (Figure 3).

After radiation, wound healing disorders are more frequent, therefore skin grafts are often not useful for defect reconstruction in such cases. Primary treatment with pedicled or microsurgical free flaps can be appropriate (52).

## Conclusion

**Figure 4:**
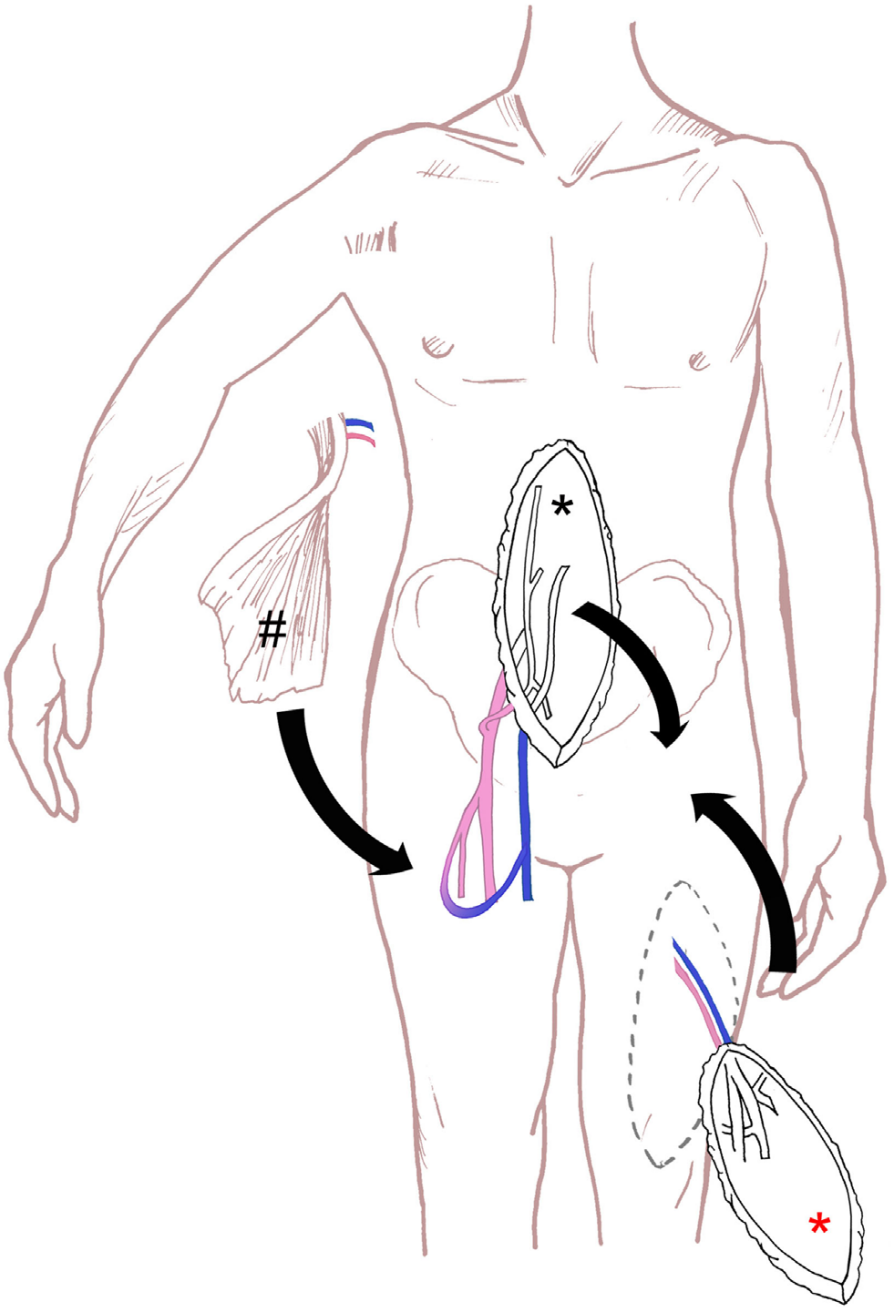
**Schematic drawing of the surgical technique for perineal defect reconstruction, with schematic drawing of VRAM flap (black asterisk), ALT flap (red asterisk) and microsurgical free latissimus dorsi flap and an arterio-venous loop (hash)**.

The evolution of more radical excisional surgery techniques resulted in an increase in large defects of the perineum. In most of the cases, primary closure of perineal defects is not possible. Skin grafts are suboptimal in the perigenital area. For intra-abdominally and/or pelvic tumors of the rectum, the anus or the female reproductive system, which were resected through an abdominally and a sacrally surgical access, simultaneous VRAM flap reconstruction is recommendable. We suggest the VRAM flap as a particularly suitable method for pelvic reconstruction in patients with advanced colorectal cancer disease requiring pelvic exenteration. In terms of soft tissue sarcoma of the pelvic/caudal abdomen/proximal thigh region, two-stage reconstructions are possible. Many factors, like size and character of the defect have to be considered, but often a pedicled flap, like ALT flap could be used in cases of sarcoma resection. Secondary sealing itself is very challenging, if chronic sacral cavities or recurrent pelvic infections are being observed. In such cases, sufficient three-dimensional defect reconstruction often is not possible without microsurgical free flaps (Figure 4).

## Conflict of Interest Statement

The authors declare that the research was conducted in the absence of any commercial or financial relationships that could be construed as a potential conflict of interest.
